# Internal Hemipelvectomy and Pelvic Reconstruction With Non-Vascularized Fibular Graft for Chondrosarcoma Ilium

**DOI:** 10.7759/cureus.16292

**Published:** 2021-07-10

**Authors:** Bikram K Kar, Sandeep Kumar Yadav, Nagaraju Venishetty, Sharath Kowshik

**Affiliations:** 1 Department of Orthopaedic Surgery, All India Institute of Medical Sciences, Raipur, Raipur, IND

**Keywords:** chondrosarcoma, hemipelvectomy, non vascularised fibular graft, pelvic reconstruction, pelvic tumor

## Abstract

Chondrosarcoma is the third most common primary malignant bone tumor. The pelvis is the most common site with iliac bone being frequently involved. Hindquarter amputation was the standard treatment for pelvic osteosarcoma for years. Resection of tumors with wide margins gained popularity with advances in the medical field. The only resection without reconstruction of the pelvis has its own demerits which paved way for methods of reconstruction. One of these is pelvic reconstruction with non-vascularised fibular graft among many other methods, which is simple, cost-effective, and has a good functional outcome. Here, we are reporting a case of exophytic chondrosarcoma of intermediate grade in ilium in a 19-year-old male for whom internal hemipelvectomy (Enneking and Dunham type 1) and pelvic reconstruction with non-vascularised fibular graft was done with the excellent functional and radiological outcome, with a two-year follow-up.

## Introduction

Chondrosarcoma accounts for 20% of malignant bone tumours [1]. After myeloma and osteosarcoma, it is the third most common primary malignant bone tumour [2]. Patients presenting with primary chondrosarcoma are usually adults and older adults between 40 and 70 years of age. Most of the chondrosarcoma are idiopathic, arise de novo as primary or secondarily from benign cartilaginous tumours like osteochondromas and enchondromas [1,3]. The most common occurrence is the pelvis with iliac bone involving more frequently followed by pubic bone and ischium. Other common sites are the proximal femur, proximal humerus, distal femur and ribs [4]. As pelvic chondrosarcoma is deeply seated, patients present with large extra skeletal mass without any specific symptoms other than pain. It needs surgical excision as chemotherapy and radiotherapy have no significant role [5]. For years hindquarters amputation was the gold standard surgery for pelvic malignancy due to the complex anatomy of the pelvis and difficulty in reconstructing significant bony defects. With advances in imaging and surgical techniques, limb salvage procedures gained popularity [2]. The goals of these surgeries are to resect lesions with wide margins without compromising the oncological outcome, minimize surgical complications and reconstruct the bony defects. Thus, where tumours can be resected with wide margins, limb-sparing internal hemipelvectomy with pelvic reconstruction becomes the optimum treatment for malignant pelvic tumours [5]. Reconstruction provides a stable pelvis and spinal column by maintaining continuity of sacrum, ilium [6]. Advantages of limb-sparing surgery are cosmetic appearance, psychological confidence, and ability to walk. For reconstruction, free vascularised fibular grafts are employed. But this required considerable time and microsurgical expertise. Studies have shown no conclusive evidence that they perform better over nonvascularized fibular graft [4]. Reconstruction with nonvascularized fibular graft is simpler, less time-consuming than with vascularised grafts and works well [5,7]. This study reports a case of chondrosarcoma ilium treated with hemipelvectomy and reconstruction of the pelvis with a non-vascularized fibular graft.

## Case presentation

**Figure 1:**
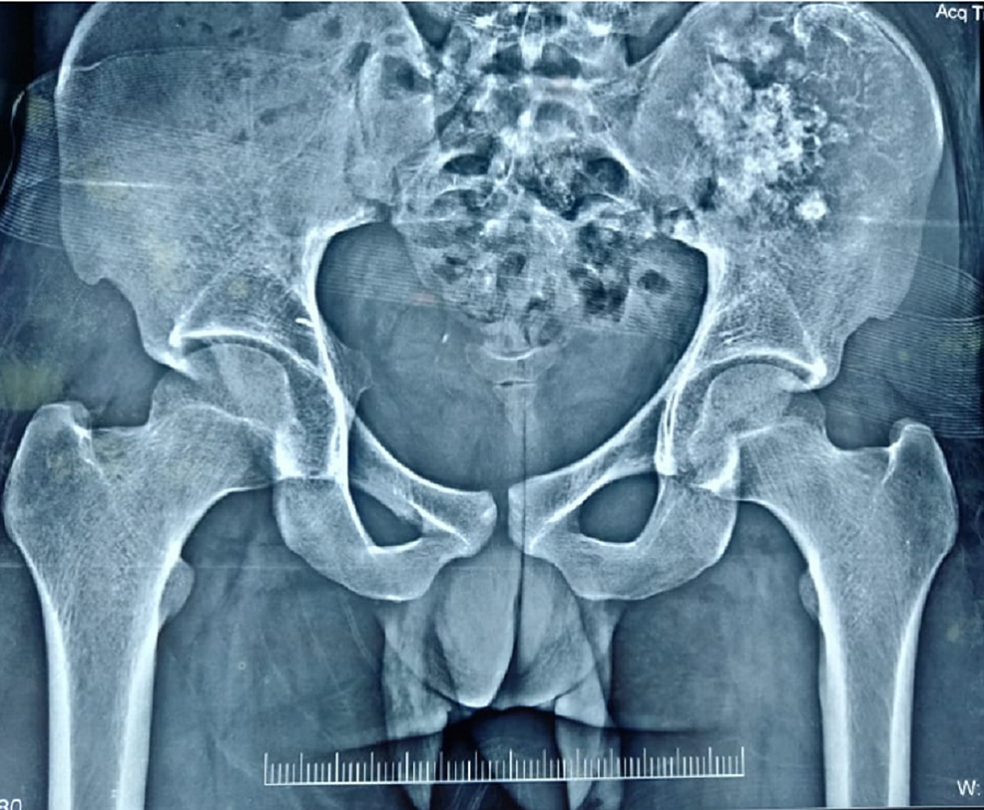
Preoperative radiograph showing popcorn calcifications on the background of lysis over the left ilium.

**Figure 2:**
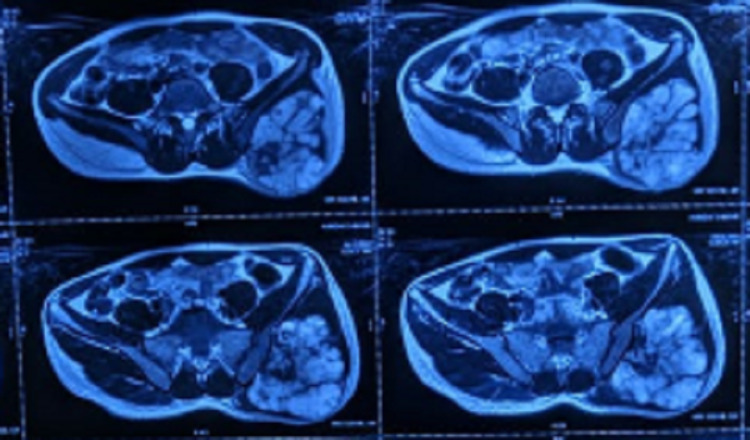
Preoperative MRI showing an exophytic tumour with heterogeneous intensity arising from the gluteal surface of the left ilium.

A 19-year-old man came in with a painful tumour over his left gluteal region that had been growing in size for the previous eight months. On examination, a lobular, firm, painful mass measuring 12 × 10 cm^2^ is palpated over the left gluteal region and fixed to the underlying ilium. There is no evidence of neurovascular involvement in the left hip, and it has a good functional range of motion. A plain radiograph of the pelvis (Figure 1) shows popcorn calcifications on the background of lysis over the left ilium, suggesting chondrosarcoma. MRI and routine blood investigations are done. An exophytic tumour with heterogeneous intensity arising from the gluteal surface of the left ilium was discovered by MRI (Figure 2), suggesting chondrosarcoma of the left ilium. Sacroiliac joint and neurovascular structures remain uninvolved. The biopsy is done by wide-bore needle, and a histopathology report confirmed the tumour as chondrosarcoma of intermediate grade. Metastatic workup showed no distant metastasis. After an anaesthesia checkup and obtaining consent, the patient operated. Through posterior skin incision, left iliac wing with tumour resected with wide margins, medially from left sacroiliac joint to laterally till 5 cm distal to tumour margin. Tumour-free margins were confirmed by intraoperative frozen section examination.

**Figure 3:**
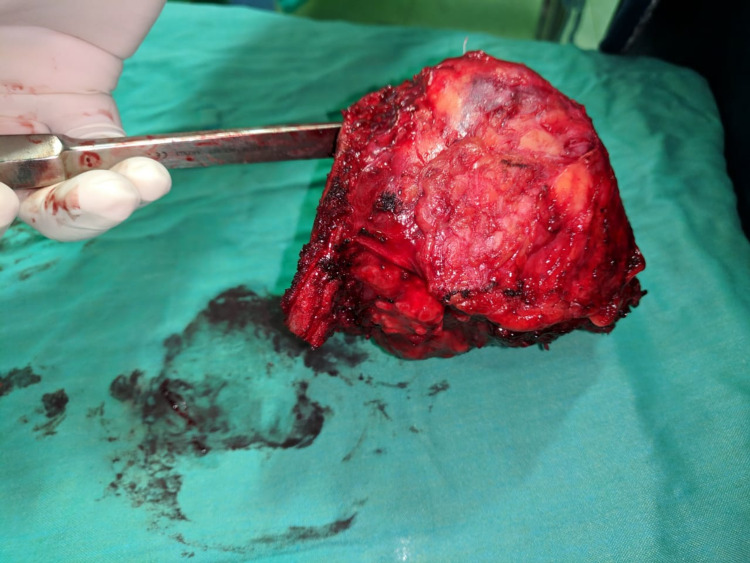
Excised tumour with encapsulating pseudo capsule.

**Figure 4:**
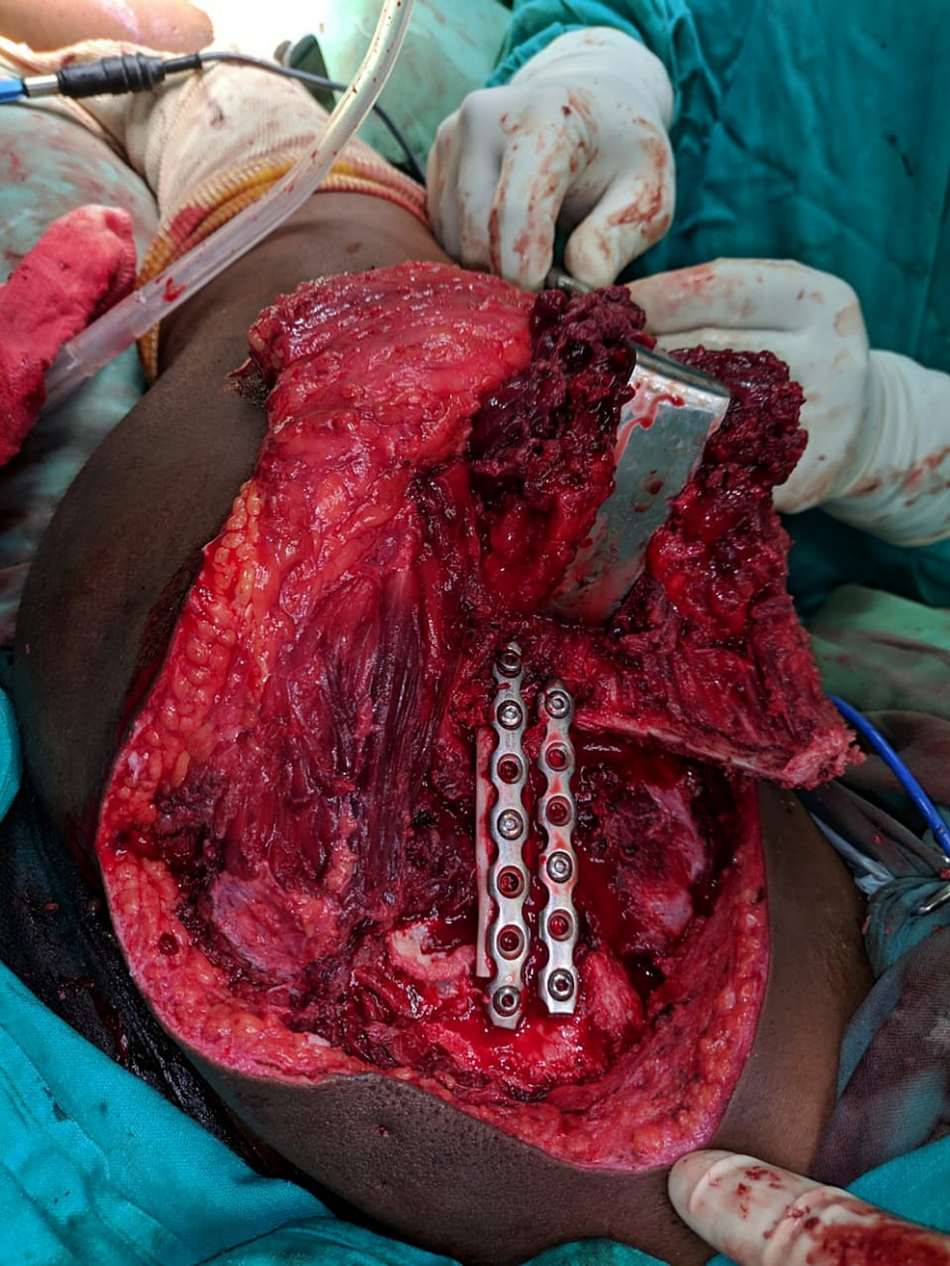
Intraoperative fixation image showing fibula graft fixed with recon plates.

**Figure 5:**
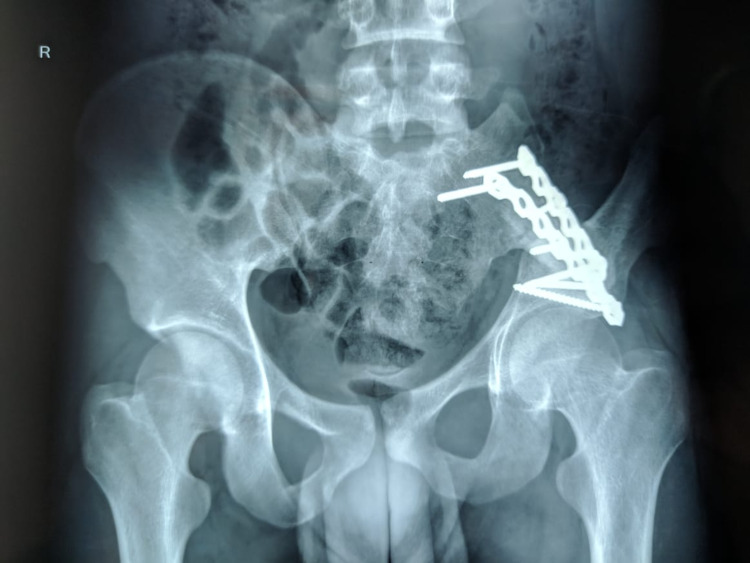
Two years postoperative radiograph showing union of graft.

**Figure 6:**
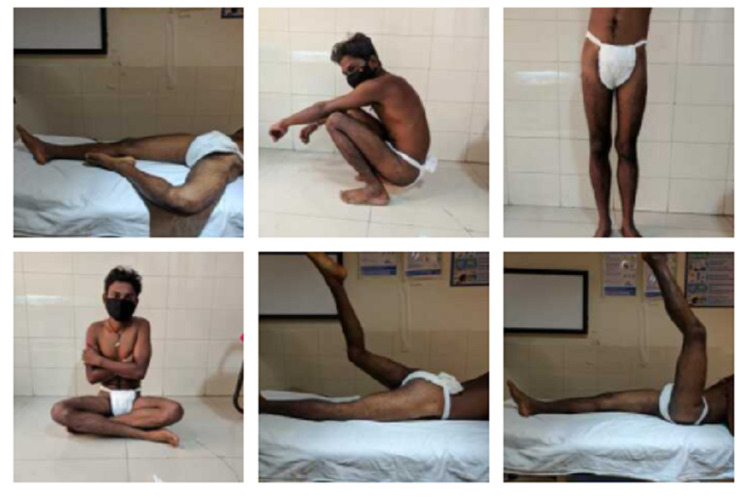
Two years postoperative clinical image showing functional status.

A 10 × 8 cm^2^ excised tumour (Figure 3) with encapsulating pseudo capsule sent to pathology for final diagnosis and free margins. Because the tumour had not invaded the neurovascular bundle or the gluteus flap, they were secured. Ipsilateral fibular strut graft harvested, and the same was split longitudinally into two and bridged between the lateral surface of sacrum and remaining iliac bone. Graft secured and fixed with recon plates (Figure 4). The gluteus flap repositioned, and the incision closed over a drain. Surgical site sutures were removed on postoperative day 12. Early in-bed exercises and hip exercises are advised. Partial weight-bearing with a walker started after one month. Full weight-bearing is suggested when the radiograph has shown graft union after three months. Two years follow-up of the patient has shown complete union of graft on radiographs (Figure 5), and clinically patient was satisfied and performed his daily activities (Figure 6).

## Discussion

Despite the advancements in surgical techniques, resection of malignant pelvic tumours is still a challenge for many surgeons. Chondrosarcoma is one of the common tumours in the pelvic region and produces a huge mass at the time of presentation. Hindquarter amputation was routine surgery for malignant pelvic tumours until Enneking advocated limb-sparing surgery, internal hemipelvectomy. Internal hemipelvectomy helps achieve local and systematic control of diseases and preserve leg and the ability to walk [8]. Complex pelvic anatomy with neurovascular structures and visceral organs makes limb-sparing surgeries difficult. Preservation of femoral nerve, artery, vein and external iliac artery and vein is important in limb-sparing surgeries for pelvic tumours [9]. Internal hemipelvectomy was first performed in the 1960s, but there were no acceptable reconstructive methods available. Further several reports of this procedure described it as an innovative technique with the reconstruction of the pelvic ring [1]. Enneking and Dunham proposed eight subtypes of hemipelvectomy based on four zones (I - ilium, II - periacetabular area, III - pubis and ischium, IV - sacrum), each requiring a different reconstruction method [4]. Reconstruction provides stability of the pelvis and spinal column by maintaining continuity of sacrum, ilium and pubis [6]. Reconstruction prevents long-term complications of the unstable pelvis, chronic pain and scoliosis. Reconstruction includes the use of prostheses, autoclave grafts, auto and allograft, autograft containing tumour treated with liquid nitrogen or other methods, vascularized or non-vascularized autologous free fibular grafts [1]. Each approach has its benefits and drawbacks, but it is widely assumed that pelvic reconstruction improves functional outcomes [10]. In reconstruction surgeries, it is vital to ensure good muscular coverage to reduce infections and for good results [11]. Reconstruction with vascularized fibular graft was first described in 1989 but has the disadvantage of more operating time for vascular anastomosis and requiring two operating teams of surgeons [10,12]. Studies have shown no conclusive evidence that they perform better over non-vascularized fibular grafts. Reconstruction with nonvascularized fibular graft is simpler, less time-consuming than with vascularized grafts and works well [7]. Preserving the periosteum of the nonvascularized fibular graft is essential for callus formation and less surgical time reduces infection rates [1]. Hillmann et al., in their study, reported that reconstruction with nonvascularized fibular graft performed better than other methods in functional outcome and complications [13]. In another study, the use of non-vascularized fibular graft in pelvic reconstruction has improved functional outcome with musculoskeletal tumour society score, comparable with vascularized graft and endoprosthesis [1]. Krieg and Hefti reported that nonvascularized fibular graft transfer in musculoskeletal tumour reconstruction is a less expensive, shorter and simple procedure than vascularised graft giving biological reconstruction with good long-term results and relatively low donor site complications [7]. Thus, the use of nonvascularized fibular graft for pelvic reconstruction has become a cost-effective, simple procedure giving the patient a stable pelvis.

## Conclusions

In selected cases, limb-sparing surgery is an effective alternative for patient psychological confidence in malignant pelvic tumour with wide resection margins. After internal hemipelvectomy, reconstruction provides stability to the pelvis, helps in rehabilitation, walking and prevents long-term complications. In our case, the use of non-vascularized fibular graft for pelvic reconstruction after resection of chondrosarcoma of ilium has given excellent functional outcomes for the patient. Hence, we propose this technique as a simple and cost-effective reconstructive surgery after hemipelvectomy in malignant pelvic tumours.
